# Down-Regulation of miR-378d Increased Rab10 Expression to Help Clearance of *Mycobacterium tuberculosis* in Macrophages

**DOI:** 10.3389/fcimb.2020.00108

**Published:** 2020-03-17

**Authors:** Yifan Zhu, Yao Xiao, Delai Kong, Han Liu, Xi Chen, Yingyu Chen, Tingting Zhu, Yongchong Peng, Wenjun Zhai, Changmin Hu, Huanchun Chen, Si Zhu Suo Lang, Aizhen Guo, Jiaqiang Niu

**Affiliations:** ^1^The State Key Laboratory of Agricultural Microbiology, Huazhong Agricultural University, Wuhan, China; ^2^College of Veterinary Medicine, Huazhong Agricultural University, Wuhan, China; ^3^Key Laboratory of Development of Veterinary Diagnostic Products, Key Laboratory of Ruminant Bio-Products of Ministry of Agriculture and Rural Affairs, Huazhong Agriculture University, Wuhan, China; ^4^Hubei International Scientific and Technological Cooperation Base of Veterinary Epidemiology, International Research Center for Animal Disease, Ministry of Science and Technology, Huazhong Agricultural University, Wuhan, China; ^5^Department of Animal Sciences, Tibet Agricultural and Animal Husbandry College, Linzhi, China

**Keywords:** *Mycobacterium tuberculosis*, microRNA-378d, macrophage, Rab10, cytokines, NF-κB

## Abstract

*Mycobacterium tuberculosis* (*M. tb*) can survive in the hostile microenvironment of cells by escaping host surveillance, but the molecular mechanisms are far from being fully understood. MicroRNAs might be involved in regulation of this intracellular process. By RNAseq of *M. tb*-infected PMA-differentiated THP-1 macrophages, we previously discovered down-regulation of miR-378d during *M. tb* infection. This study aimed to investigate the roles of miR-378d in *M. tb* infection of THP-1 cells by using a miR-378d mimic and inhibitor. First, *M. tb* infection was confirmed to decrease miR-378d expression in THP-1 and Raw 264.7 macrophages. Then, it was demonstrated that miR-378d mimic promoted, while its inhibitor decreased, *M. tb* survival in THP-1 cells. Further, the miR-378d mimic suppressed, while its inhibitor enhanced the protein production of IL-1β, TNF-α, IL-6, and Rab10 expression. By using siRNA of Rab10 (siRab10) to knock-down the Rab10 gene in THP-1 with or without miR-378d inhibitor transfection, Rab10 was determined to be a miR-378d target during *M. tb* infection. In addition, a dual luciferase reporter assay with the Rab10 wild-type sequence and mutant for miR-378d binding sites confirmed Rab10 as the target of miR-378d associated with *M. tb* infection. The involvement of four signal pathways NF-κB, P38, JNK, and ERK in miR-378d regulation was determined by detecting the effect of their respective inhibitors on miR-378d expression, and miR-378d inhibitor on activation of these four signal pathways. As a result, activation of the NF-κB signaling pathway was associated with the down-regulation of miR-378d. In conclusion, during *M. tb* infection of macrophages, miR-378d was down-regulated and functioned on decreasing *M. tb* intracellular survival by targeting Rab10 and the process was regulated by activation of the NF-κB and induction of pro-inflammatory cytokines IL-1β, TNF-α, IL-6. These findings shed light on further understanding the defense mechanisms in macrophages against *M. tb* infection.

## Introduction

*Mycobacterium tuberculosis* (*M. tb*) is the etiologic agent of tuberculosis (TB). In 2018, TB caused an estimated 1.2 million deaths and 10 million new cases (World Health, 2019). As a pathogen transmitted by aerosol, *M. tb* was first recognized to undergo phagocytosis by alveolar macrophages inside the lungs. After engulfment, the bacilli can persist within host cells of granulomas for its survival and lead to a latent phase. Macrophages are major host cells for M. tb intracellular growth and its persistence during all phases of TB and contribute to stimulation of both innate and acquired immune responses, therefore playing an essential role in protection against the infection (Rajaram et al., 2014).

MicroRNAs (miRNAs) belong to a class of small non-coding conservative RNAs within eukaryotic species. They usually function as critical regulators of gene expression through post-transcriptional modification of its target mRNAs for translation inhibition or for degradation (Bartel, 2004). These are realized by their binding of the miRNA-recognition sequences within the 3′-untranslated region (3′-UTR) of corresponding targeting mRNAs to their miRNA “seed” region between 2 and 8 nucleotides at the 5′ end (Xie et al., 2005; Etna et al., 2018). MiRNAs can participate in host immune response (Zhang et al., 2014; Liu et al., 2016) and regulate cellular processes, such as cytokine production, autophagy, and apoptosis (Bartel, 2004; Cheloufi et al., 2010; Stanley et al., 2014; Wang et al., 2015; Kim et al., 2017; Ma et al., 2018; Yang and Ge, 2018). For example, many studies have reported that miR-378 plays an important role in different types of cancer. It acts as a tumor suppressor to inhibit the growth and proliferation of tumor cells in hepatocellular carcinoma (Zeng et al., 2017); however, in liver cancer cells, over-expression of miR-378 enhances cell migration and metastasis by down-regulating Fus expression (Ma et al., 2014). miR-378 belongs to a large family of evolutionarily conserved miRNAs with eight members (miR-378 a/b/c/d/e/f/h/i). Because they share similar seed sequences, a sequence of 6–8 nucleotides that is most critical for mRNA target recognition, they are believed to have similar activities and targets (Ganesan et al., 2013). Importantly, a more recent paper about esophageal squamous cell carcinoma reported that miR-378a-3p targeted Rab10 during the process of this cancer development (Ding et al., 2018).

Rab family members are a group of small GTPases. They constitute the largest branch of the Ras superfamily (Alix et al., 2011), and regulate eukaryotic membrane trafficking in both endocytic pathway and exocytic pathway, while their functions are limited to particular intracellular transport steps (Hutagalung and Novick, 2011; Pfeffer, 2013; Chua and Tang, 2018). Rab10 is a member of Ras oncogene family, which plays a role in pathogen infection. It has been shown that Rab10 is vital for optimal macrophage activation (Wang et al., 2010) and bactericidal activity in macrophages (Liu et al., 2017) by promoting phagolysosome fusion as a membrane-trafficking regulator (Zerial and McBride, 2001). On the other hand, in LPS treated macrophages, Rab10 silencing could decrease TLR4 expression on cellular surface, and inhibit activation of signaling pathways IRF3, MAPK, and NF-κB, and reduce generation of IFN-β, IL-6, and TNF-α by the cells (Wang et al., 2010).

According to previous reports, at least 30 miRNAs may be involved in *M. tb* infection, and at least 10 of these might be associated with the survival of *M. tb* in macrophages. MiR-27a expression was up-regulated, and expression of its target protein, CACNA2D3, an ER-located Ca^2+^ transporter, was reduced during *M. tb* infection leading to inhibition of autophagosome formation (Liu et al., 2018). The miR-33 locus was induced by *M. tb* infection to reprogram autophagy and host lipid metabolism and enable *M. tb* to survive intracellularly and persist in the hosts (Ouimet et al., 2016). More recently, Kumar et al. found that *M. tb* inhibits miR-let-7f expression, which led to suppression of the inflammatory response and nitric oxide production via target A20, an inhibitor of NF-κB signaling (Kumar et al., 2015). Our previous results regarding the transcriptome of infected THP-1 macrophages obtained by RNA-seq showed that *M. tb* infection induced differential miRNA expression profiles, revealing hundreds of novels and known miRNAs, including miR-378d, a member of miR-378 family (data not shown). However, to our knowledge, there is no published report so far on the role of miR-378d in *M. tb* infection or relationship between miR-378d and Rab10 during *M. tb* infection.

This study was aimed to investigate the functions of miR-378d in *M. tb* infection and the related mechanism. The results showed that down-regulation of miR-378d induced by *M. tb* infection enhanced expression of its target Rab10, activation of NF-κB signaling and increased the generation of some inflammatory cytokines which might contribute to decrease of *M. tb* survival in THP-1 cells.

## Materials and Methods

### Bacterial Strains and Cell Culture

The culture of *M. tb* and infected cells was performed in the facility at animal biosafety level-3 (ABSL-3) in Huazhong Agricultural University, Wuhan, China. The *M. tb* 1458 strain (clinic strain isolated from diseased cattle with TB) (GenBank accession no. GCA_001855255.1) was grown broth medium Middlebrook 7H9 with 10% of oleic acid-albumin-dextrose-catalase (OADC) (BD PharMingen, USA) and 0.05% Tween-80 (Sigma, USA) at 37°C. For infection, the bacteria were cultured for 10–20 days, until the broth began to become turbid, and then subjected to centrifugation at 3,000 × g for 10 min to precipitate the bacteria, the pellet was then re-suspended in above broth medium at 1/2 of original volume. The suspension was dispersed by passing through an insulin syringe for five times, and standing for 10 min. Bacterial number was estimated according to the formula of 0.6 OD_600nm_ equaling to about 1 × 10^8^ bacteria/mL (Karim et al., 2011). Meanwhile 100 μL of the suspension were taken to be 10-fold serially diluted, and 50 μL for each dilution were cultured on 10% OADC Middlebrook 7H11 agar plate (BD PharMingen, USA) and incubated at 37°C for 14 d −21 d to count viable bacteria (CFU/mL).

The THP-1 cell line (ATCC® TIB-202TM), was cultured in complete RPMI-1640 (Hyclone, USA) with 10% FBS (Gibco, USA). Before infection, THP-1 was pre-treated in culture medium including 40 ng/mL phorbol 12-myristate 13-acetate (PMA) (Sigma, USA) for 24 h at 37°C, 5% CO_2_ to be differentiated into human macrophages. Other cell lines including the Raw 264.7 (the murine macrophage cell line) (ATCC® TIB-71TM), and 293T (a cell line of human embryonic kidney Cells) (ATCC® CRL-3216™) were grown in 10% FBS-DMEM medium (Hyclone, USA).

### Cell Infection With *M. tb*

PMA-differentiated THP-1 cells and Raw 264.7 cells were infected with *M. tb* 1458 (MOI = 10) (Kumar et al., 2015) and incubated for 12 h (defined as −12 h) to enable M. tb to sufficiently enter the cells. Then, the cells were washed two times with fresh medium to remove extracellular *M. tb* (Kumar et al., 2015). By taking this time point as 0 h, the cells continued to be cultured in complete medium with 100 μg/mL gentamicin for various times (6, 12, and 24 h). The cells and supernatants were collected by centrifugation for further analysis.

### Cell Transfection

The miR-378d mimic, miR-378d inhibitor, siRab10, and their control sequences were synthesized by a commercial company (GenePharma, China) (Table 1).

**Table 1:** siRNA and RNA oligonucleotides sequences.

**Sequence name**	**Oligonucleotide sequence (5^′^ → 3^′^)**
siRab10	guide: GGGUAUCAUGCUAGUAUAUTT
	passenger: AUAUACUAGCAUGAUACCCTT
siRab10 negative control	UUCUCCGAACGUGUCACGUTT
	ACGUGACACGUUCGGAGAATT
Hsa-miR-378d mimic	guide: ACUGGACUUGGAGUCAGAAA
	passenger: UCUGACUCCAAGUCCAGUUU
Hsa-miR-378d mimic negative control	UUCUCCGAACGUGUCACGUTT
	ACGUGACACGUUCGGAGAATT
Hsa-miR-378d inhibitor	UUUCUGACUCCAAGUCCAGU
Hsa-miR-378d inhibitor negative control	CAGUACUUUUGUGUAGUACAA

THP-1 cells (5.0 × 10^5^) were mixed with siRNA/miRNA transfection INTERFERin® reagents (Ployplus, France) and various synthesized RNA fragments including miR-378d mimic, miR-378d inhibitor, siRab10, or three types of negative control agents, each at the concentration of 15 pg/well (equal to 15 nM which was determined in advance from concentrations of 5, 10, 15, 20, 25, and 30 nM according to the criterion that was the highest level without cytotoxicity) by pipetting 10 times, respectively, and then seeded into a 12-well plate for 24 h. Then, the transfected THP-1 cells were incubated with PMA for 12 h to differentiate into macrophages, and infection was performed as described above.

For co-transfection, the cells were divided into four groups and, respectively treated as follows: **Group 1** was transfected with the inhibitor negative control and siRab10 negative control, **Group 2** with inhibitor negative control and siRab10, **Group 3** with miR-378d inhibitor and siRab10 negative control, and **Group 4** with miR-378d inhibitor and siRab10. Each reagent was equally added at 15 pg/well (15 nM).

### Quantitative Real-Time PCR (qRT-PCR)

Quantitative real-time PCR (qRT-PCR) was used to detect the relative transcription of the related genes in different experiments.

The general protocol was as follows: Total RNAs were extracted from PMA-differentiated THP-1 cells infected with live or treated with heat-inactivated (HI) (95°C, 30 min) *M. tb* at indicated time points (0, 6, 12, and 24 h) using TRIzol reagent (Invitrogen, USA) following the manufacturer's instructions. Uninfected cells were used as control. The concentration of total RNA was measured by Ultraviolet Spectrophotometer at 260 nm (Thermo, USA). To monitor the levels of miRNA and mRNA, cDNA was synthesized from 1 μg of total RNA using an All-in-one TM miRNA qRT-PCR Detection Kit (GeneCopoeia, USA) or from 500 ng of total RNA using a HiScript® II Q RT SuperMix (Vazyme, China), respectively. The expression of miRNA and mRNA was transferred into relative expression by setting the uninfected control as 1.

After reverse transcription, qRT-PCR analysis was performed using the AceQ qPCR SYBR Green master mix (Vazyme, China) on an ABI ViiA 7 Real-Time PCR System (ABI, USA). The relative expression of miRNA was normalized to U6, while that of mRNA was normalized to β-actin, respectively. The gene-specific primers (Quintara Biosciences, China) for qRT-PCR used in this study are listed in Table 2. Data were presented using 2^−ΔΔCt^. All experiments were performed in triplicate.

**Table 2:** Primers for qRT-PCR and PCR to generate Rab10 3′-UTR and its mutant.

**Primer name**	**Sequence (5^′^ → 3^′^)**	**Products (bp)**	**Application**	**References**
Universal reverse	GCTGTCAACGATACGCTACGTAAC		qRT-PCR	Etna et al., 2018
U6	F: CTCGCTTCGGCAGCACA			Li et al., 2016
	R: AACGCTTCACGAATTTGCGT			
hsa-miR-378d	ACTGGACTTGGAGTCAGAAA			
mmu-miR-378d	ACTGGCCTTGGAGTCAGAAGGT			
hsa-β-actin	F: CATGTACGTTGCTATCCAGGC	250		Ren et al., 2016
	R: CTCCTTAATGTCACGCACGAT			
hsa-IL-1β	F: GTGGCAATGAGGATGACTTGTTC	120		
	R: GGTGGTCGGAGATTCGTAGCT			
hsa-IL-6	F: ACTCACCTCTTCAGAACGAA	149		Shen et al., 2015
	R: CCATCTTTGGAAGGTTCAGG			
hsa-TNF-α	F: GGAGAAGGGTGACCGACTCA	70		
	R: CTGCCCAGACTCGGCAA			
hsa-Rab10	F: TGGAACTACAAGGAAAGAAGAT	78		Liu et al., 2017
	R: TAGTAGGAGGTTGTGATGGT			
Rab10 3′-UTR WT	F:CCTCGAGTCATCTTAACTATCCAAGCCA	526	generation of the Rab10 3′-UTR	
	R:TTGCGGCCGCGAAAATTACAGCCAAGCAA			
Rab10 3′-UTR Mut	F:CAGTTGTATTATTCTGGACATCTTATCAACATTA	526		
	R:TAATGTTGATAAGATGTCCAGAATAATACAACTG			

The miR-378d expression was first detected in *M. tb*-infected and uninfected, PMA-differentiated THP-1 cells and Raw 264.7 cells at 6, 12, and 24 h post infection (PI).

Since NF-κB and MAPK signaling pathways were previously reported to mediate the survival of *M. tb* in macrophages by mechanisms such as induction of pro- and anti-inflammatory cytokine secretion during mycobacterial infection (Chan et al., 2001; Bai et al., 2013; Xu et al., 2014), we tested whether these signaling pathways might modify miR-378d expression during *M. tb* infection. PMA-differentiated THP-1 cells were treated with inhibitors (purchased from Target Mol, USA) of the ERK (10 μM U-0126), JNK (10 μM SP600125), p38 (10 μM SB203580), or NF-κB (20 μM SC514) pathways, respectively, for 2 h and infected with M. tb as previously described. At 24 h PI, the relative expression of miR-378d was measured. In addition, gene expression of the cytokines IL-1β, IL-6, and TNF-α and Rab10 was detected when miR-378d was over-expressed by transfecting miR-378d mimic or down-regulated by transfecting miR-378d inhibitor in PMA-differentiated THP-1 macrophages.

### Western-Blot Analysis

Western-blot analysis was used to detect gene expression at the protein level of critical molecules for four signaling pathways and Rab10, when miR-378d was over-expressed by transfecting miR-378d mimic or down-regulated by transfecting miR-378d inhibitor in PMA-differentiated THP-1 macrophages.

The infected cells were lysed with lysis buffer (Sigma, USA). Lysates were separated by 10% SDS-PAGE gel and transferred to a polyvinylidene difluoride (PVDF) membrane (Millipore, USA). The membranes were blocked with 5% BSA in TBST (TBS containing Tween 20) and then incubated at 4°C overnight with the primary antibodies: mouse mAb to NF-κB p65(L8F6) (Cat no. 6956), mouse mAb to Phosphor-NF-κB p65 (Cat no. 3036), rabbit mAb to p38 MAPK (Cat no. 8690), rabbit mAb to Phospho-p38 MAPK (Cat no. 4511), rabbit mAb to p44/42 MAPK (Erk1/2) (Cat no. 4695), rabbit mAb to Phospho-p44/42 MAPK (Erk1/2) (Cat no. 4370), rabbit mAb to JNK MAPK (Cat no. 9252), rabbit mAb to Phospho-JNK MAPK (Cat no.4668) (Cell Signaling Technology, USA), mouse polyclonal antibodies to β-actin (Protech, China, Cat no. 60008-1), rabbit mAb to Rab10 (Abcam, USA, Cat no.181367) at 1:1000 dilution in TBST solution. The protein band intensities were measured using Western Bright ECL (Advansta, USA), and β-actin was used as an internal reference. Quantitative analysis of bands was performed using ImageJ software (National Institutes of Health, USA).

### Enzyme-Linked Immunosorbent Assay (ELISA) of Cytokines

THP-1 cells (5.0 × 10^5^) were transfected with oligonucleotides in a 12-well plate for 24 h and were incubated with *M. tb* 1458 strain (MOI = 10) for 12 h after treatment with PMA for 12 h. Then culture supernatants collected at indicated time points were detected for the levels of cytokines IL-1β, IL-6, and TNF-α by ELISA (Neobioscience, China) according to the products' instructions. Standard curves in each test were used to calculate cytokine concentrations.

### Bacterial Plate Assay to Count Colony-Forming Units (CFU)

The infected cells were lysed at indicated time points using sterile deionized water, and viable bacilli were enumerated by plating of serially-diluted lysates (as described above) on 7H11 agar plates supplemented with 10% OADC and incubated at 37°C for 15–21 d. Each sample was plated in triplicate.

### Determination of Interaction Between miR-378d and Rab10 3′-UTR With Dual Luciferase Reporter Assay

To test specific interaction of miR-378d and Rab10 3′-UTR, the dual luciferase reporter assay was performed. Firstly, the luciferase reporter plasmid psiCHECK-2 (Promega, USA) was used to construct the recombinant plasmids containing Rab10 3′-UTR or the mutated sequence. A 0.5-kb region of the human Rab10 3′-UTR (named Rab10-WT) containing the predicted miRNA miR-378d-binding sites was amplified from the THP-1 genome with the primer hsa-Rab10 by conventional PCR and was cloned into the psiCHECK-2 luciferase reporter plasmid (named psiCHECK-Rab10-WT) (Promega, USA). Then, the Rab10 3′-UTR mutant (named Rab10-Mut) from the Rab10 3′-UTR WT fragment was amplified by using overlapped extension PCR for site-directed mutation of five discrete nucleotides in the predicted binding site of Rab10 3′-UTR to miR-378d (Vallejo et al., 2008) and further cloned into the psiCHECK-2 luciferase reporter plasmid (named psiCHECK-Rab10-Mut). The primers are listed in Table 2.

0.5 μg each of psiCHECK-Rab10-WT and psiCHECK-Rab10-Mut were co-transfected with 15 pg/well (15 nM) each of the miR-378d mimic or control mimic into 5 × 10^5^ HEK 293T cells in 1 ml medium grown in a 24-well cell culture plate using Lipofectamine 2000 (Invitrogen,USA). At 24 h post transfection, the Renilla and Firefly luciferase activities of the cells were tested by using Dual-Luciferase Reporter® Assay system (Promega, USA) according to the product's instruction, and luminescence intensity was measured by a multimode microplate reader (BioTek Synergy 2, USA). The activity ratio of Renilla reniformis luciferase to Firefly luciferase defined as the relative luciferase activity was calculated.

### Prediction Analysis of miR-378d Targets

For the identification of putative target mRNAs of miR-378d, several prediction algorithms, namely To predict potential target mRNAs of miR-378d, several predictive algorithms, including microRNA (http://www.microrna.org/microrna/getdownloads.do), Targetscans7.1 (http://www.targetscan.org), miRBD (http://mirdb.org/miRDB/), and miRecords (http://c1.accurascience.com/miRecords/) were adopted to analyze the conservation, complementarity, and thermodynamic stability between the 3′-UTR of mRNA and the 5′ terminal of the miR-378d seed sequence. All four prediction algorithms considered the predicted genes to be candidate miR-378d targets. The cluster and path analysis was performed using KEGG PATHWAY Database (http://www.kegg.jp/kegg/) and DAVID Bioinformatics Resources 6.8 (https://david.ncifcrf.gov/).

### Statistical Analysis

GraphPad Prism 7.0 built-in Softwares (La Jolla, CA, USA) was used in statistical analysis. All of the results are shown as the mean ± SD. The unpaired two-tailed Student *t*-test or one-way analysis of variance (ANOVA) was conducted when it is necessary. Statistical difference was considered to be significant when *p* < 0.05 and the *p* < 0.05, < 0.01, < 0.001 were, respectively indicated as ^*^, ^**^, and ^***^ in figures.

## Results

### *M. tb* Infection Down-Regulates miR-378d in Macrophages

**Figure 1:**
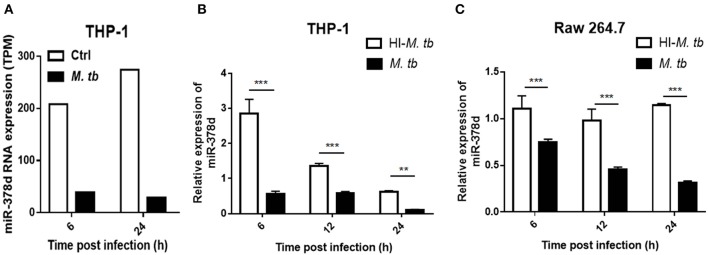
*M. tb* infection down-regulated miR-378d expression in macrophages. PMA-differentiated THP-1 cells and RAW 264.7 cells were infected with live (*M. tb*) or treated with heat-inactivated (HI-*M. tb*) *M. tb* at a MOI of 10 for 12 h, or mock-treated with PBS (ctrl). RNA were extracted from the cells, and miR-378d expression was detected by RNA-seq or qRT-PCR at indicated time points. **(A)** The miR-378d expression valued by trans Per Million (TPM) was detected by RNA-seq in the cells infected with *M. tb* at 6 and 24 h post infection as compared to an uninfected control (ctrl). **(B)** Confirmation of miR-378d expression by qRT-PCR in THP-1 macrophages infected with *M. tb* at 6, 12, and 24 h post infection as compared to HI-*M. tb-*treated cells. **(C)** The confirmation of miR-378d expression by qRT-PCR in RAW264.7 macrophages infected with *M. tb* at 6, 12, and 24 h post infection as compared to HI-*M. tb*-treated cells. This figure is one representative of three independent experiments. The values of *p* < 0.01 and 0.001 are marked by ** and ***, respectively.

Previous data from this lab from a RNA-seq study discovered that miR-378d was significantly down-regulated in *M. tb*-infected, PMA-differentiated THP-1 cells at 6 and 24 h post infection (PI) (Figure 1A). To confirm this finding, miR-378d expression in PMA-differentiated THP-1 cells was detected by qRT-PCR and compared between cells infected with live *M. tb* infection and those treated with heat-killed *M. tb* by defining the uninfected control as 1. The miR-378d expression in live *M. tb*-infected cells was significantly lower than in the heat-killed *M. tb* control group (*p* < 0.01) at three time points (6, 12, and 24 h PI) in THP-1 macrophages (Figure 1B) or Raw 264.7 cells (Figure 1C). These data indicated that live *M. tb* consistently results in a decrease in miR-378d expression in infected macrophages (Figure 1).

### Upregulation of miR-378d Promotes *M. tb* Survival in Macrophages

**Figure 2:**
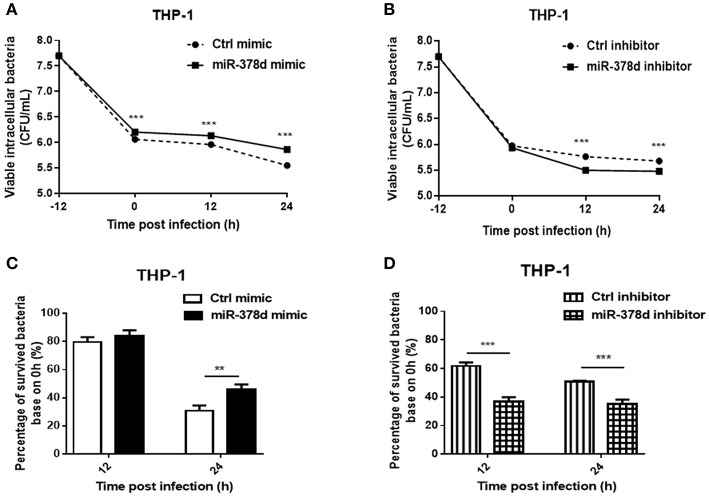
miR-378d promoted *M. tb* survival in macrophages. THP-1 cells were transfected with miR-378d mimic, inhibitor, or the corresponding negative controls for 24 h, respectively, treated with PMA for 12 h, and then infected with *M. tb* at a MOI of 10 for 12 h (defined as 0 h PI). Then the extracellular *M. tb* were washed away twice, followed by incubation with THP-1 macrophages for 12 and 24 h. Viable intracellular bacteria were determined by 7H11 agar plating assay at 0, 12, and 24 h PI. **(A)** Viable intracellular bacteria in the miR-378d mimic-treated cells (solid line) and mimic negative control (dashed line) at indicated time points. **(B)** Viable intracellular bacteria in miR-378d inhibitor-treated cells (solid line) and inhibitor negative control-treated cells (dashed line) at indicated time points. **(C)** The percentage change of surviving intracellular bacteria from time 0 between the miR-378d mimic and the negative control derived from **(A)**. **(D)** The percentage change of surviving intracellular bacteria from time 0 between the miR-378d inhibitor and its negative control derived from **(B)**. This figure is a representative of three independent experiments. The values of *p* < 0.01 and 0.001 are marked by ** and ***, respectively.

To investigate the role of miR-378d during *M. tb* infection, THP-1 cells were transfected with miR-378d mimic, inhibitor, and their negative controls for 24 h, respectively, treated with PMA for 12 h, and then infected at MOI = 10 at −12 h. The viable intracellular *M. tb* for all groups were counted (CFU/mL) at 0, 12, and 24 h PI and compared (Figures 2A,B). At all three time points, the number of intracellular *M. tb* in the mimic group was significantly higher than the control mimic (*p* < 0.001) (Figure 2A). In contrast, CFU counts in miR-378d inhibitor group was significantly less than the control group at 12 and 24 h PI (*p* > 0.001), but no difference at 0 h indicating the time dependent manner (Figure 2B).

In addition, the proportions (%) of intracellular *M. tb* at 12 and 24 h to that at 0 h were calculated, respectively and the decrease trend in bacterial number was compared (Figures 2C,D). At 12 h PI, the miR-378d mimic and control groups showed similarly decreased trends (*p* > 0.05); but 24 h PI, the percentage change in the miR-378d mimic group was significantly higher than its negative control, indicating that the miR-378d mimic promoted survival of intracellular *M. tb* (*p* < 0.001) (Figure 2C). To be contrary, the percentage changes of survived intracellular bacteria in the miR-378d inhibitor group were significantly lower than its negative control at both 12 and 24 h PI (*p* < 0.001) (Figure 2D). Together, our data demonstrated that upregulation of miR-378d promotes *M. tb* survival in macrophages, while downregulation of miR-378d suppressed *M. tb* survival in macrophages.

### miR-378d Is Regulated by Signaling Molecules in the NF-κB Pathway

**Figure 3:**
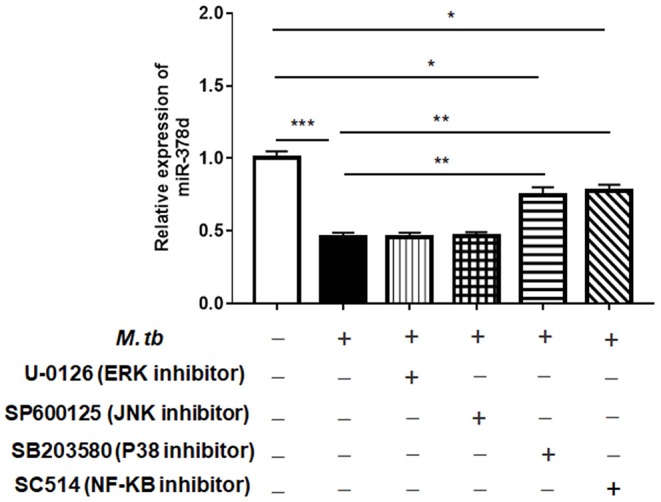
Detection by qRT-PCR of signaling pathways possibly involved in regulating miR-378d expression in *M. tb*-infected THP-1 macrophages. PMA-differentiated THP-1 macrophages were treated with inhibitors of ERK (U-0126), JNK (SP600125), p38 (SB203580), and NF-κB (SC514) pathways, respectively, for 2 h, and then infected with *M. tb* at a MOI of 10. At 24 h PI, miR-378d expression was detected by qRT-PCR. This represents one of three experiments. The *p* < 0.05, 0.01, and 0.001 values were marked by *, **, and ***, respectively.

To investigate the signaling pathways that might modify miR-378d expression after *M. tb* infection, the PMA-differentiated THP-1 macrophages were pre-treated with the four inhibitors of the ERK (U-0126), JNK (SP600125), p38 (SB203580), or NF-κB (SC514) pathways for 2 h, followed by infection with *M. tb* and measurement of the relative expression of miR-378d at 24 h PI. The results showed that compared to the negative control, the decrease in miR-378d expression due to *M. tb* infection was significantly recovered, when the infected cells were pre-treated with either NF-κB or p38 inhibitor (*p* < 0.01) as opposed to ERK or JNK inhibitors (Figure 3). However, the expression recovery by both p38 and NF-κB treatment did not reach the level of the uninfected control (*p* < 0.05), which also suggests that the p38 and NF-κB pathways partially contribute to down-regulation of miR-378d expression by *M. tb* infection.

**Figure 4:**
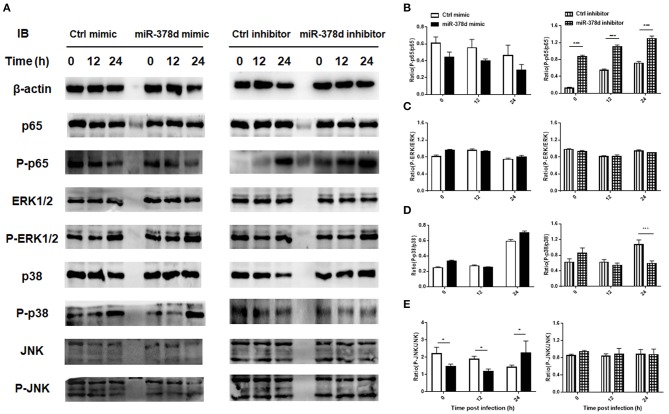
Expression and activation of the molecules critical to signaling pathways potentially regulating miR-378d expression in *M. tb*-infected macrophages. THP-1 cells were transfected with miR-378d mimic, inhibitor, or unrelated miRNA controls for 24 h, treated with PMA for 12 h, respectively, and then infected with *M. tb* at a MOI of 10. **(A)** The expression of signaling molecules p65, P-p65, p38, P-p38, ERK1/2, P-ERK1/2, JNK, and P-JNK in the infected cells was detected by Western blot assay. **(B–E)** The right panels represent the ratio of phosphorylated to unphosphorylated molecules represented by the ratio of the intensity of bands (left panel) quantified by using ImageJ and plotted using GraphPad Prism. **(B)** P-p65/p65. **(C)** P-ERK/ERK. **(D)** P-p38/p38. **(E)** P-JNK/JNK. These graphs represent one of three independent experiments. The values of *p* < 0.05 and 0.001 are marked by * and ***, respectively.

Further, the THP-1 cells were individually transfected with miR-378d mimic, inhibitor, and the control mimic or inhibitor for 24 h, respectively, and then treated with PMA for 12 h and infected with *M. tb*. At time points of 0 h, 12 h, and 24 h post infection, the expression of P-p65/p65, P-ERK/ ERK, P-p38/ p38, and P-JNK/JNK was examined by Western blot assay (Figure 4A). As a result, the ratio of expressed P-p65/p65 was significantly increased at all three time points in miR-378d inhibitor-treated cells compared to the inhibitor control cells (*p* < 0.001) (Figure 4B, right panel). Accordingly, the ratio of P-p65/p65 was similarly decreased at all three time points in miR-378d mimic-treated cells compared to the mimic control, but the difference was not significant (Figure 4B, left panel). The ratio of P-p38/p38 significantly decreased at 24 h PI (Figure 4D, right panel) in miR-378d inhibitor-treated cells compared to the control cells (*p* < 0.001). For the JNK pathway, the ratio of P-JNK/JNK was significantly decreased at 0 and 6 h PI (*p* < 0.05) but significantly increased at 24 h PI (*p* < 0.05) in the cells treated with the miR-378d mimic compared to the mimic control (Figure 4E). In contrast, there was no significant difference in other treatments or at other time points (Figure 4C).

Altogether, since inhibition of the NF-κB signaling pathway increased miR-378d expression, while suppression of miR-378d expression activated NF-κB signaling pathway as shown by consistently enhanced ratio of P-p65/p65, activation of the NF-κB signaling pathway was negatively correlated with miR-378d expression.

### miR-378d Inhibited Cytokine Expression

**Figure 5:**
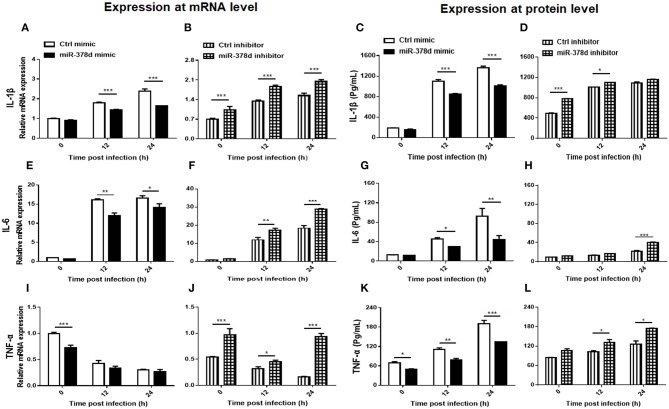
miR-378d inhibited pro-inflammatory cytokine expression. THP-1 cells were transfected with miR-378d mimic, inhibitor, or unrelated miRNA controls for 24 h, respectively, then treated with PMA for 12 h, and infected with *M. tb* at MOI of 10. The supernatants of infected cells were collected at 0 h, 12 h, and 24 h and detected. Relative mRNA expression of IL-1β **(A,B)**, IL-6 **(E,F)**, and TNF-α **(I,J)** was detected by qRT-PCR, while protein concentrations were detected with commercial ELISA kits for IL-1β **(C,D)**, IL-6 **(G,H)**, and TNF-α **(K,L)**. These graphs represent one of three independent experiments. The *p* < 0.05, 0.01, and 0.001 values were marked by *, **, and ***, respectively.

The relationship between miR-378d and the expression of cytokines (IL-1β, IL-6 and TNF-α) during *M. tb* infection was examined at the mRNA level by using qRT-PCR and the protein level by ELISA. As shown in Figure 5, the expression of these cytokines was inhibited by the miR-378d mimic but increased by the miR-378d inhibitor. Specifically, IL-1β was abundantly expressed with the highest concentration over 1,000 pg/ml, while IL-6 was weakly expressed with the highest concentration lower than 105 pg/ml.

For IL-1β, compared to the negative control, the miR-378d mimic significantly decreased IL-1β expression in transfected THP-1 cells at 12 and 24 h PI at both the mRNA and protein levels (*p* < 0.001). On the contrary, the miR-378d inhibitor increased IL-1β expression. The difference between the experimental and control groups was significant at three time points at the mRNA level, but only at 0 h (*p* < 0.001) and 12 h (*p* < 0.05) at protein level (Figures 5A–D).

For IL-6, compared to the negative control, the miR-378d mimic significantly inhibited IL-6 expression in transfected THP-1 cells at 12 and 24 h PI at both the mRNA and protein levels (*p* < 0.001). On the contrary, the miR-378d inhibitor significantly increased IL-6 expression at the mRNA level at 12 h (*p* < 0.01) and 24 h PI (*p* < 0.001), but only at 24 h (*p* < 0.001) at the protein level (Figures 5E–H).

For TNF-α, compared to the negative control, the same suppressive effect of the miR-378d mimic on TNF-α expression in transfected THP-1 cells occurred at both mRNA and protein levels, but the difference was significant only at 0 h PI for mRNA level and at all three time points for protein. On the contrary, miR-378d inhibitor significantly increased TNF-α expression at the mRNA level at all three time points [0 h (*p* < 0.001), 12 h (*p* < 0.05), and 24 h (*p* < 0.001)], but at the protein level, only at 12 and 24 h (*p* < 0.05) (Figures 5I–L).

Collectively, miR-378d inhibited expression of the cytokines IL-1β, IL-6, and TNF-α.

### miR-378d Targeted Rab10

Bioinformatic analysis using the four online prediction algorithms mentioned previously was first conducted to predict the genes targeted by miR-378d. As a result, a total of 13 potential target genes were identified (Figure S1). Coincidently, a recent study showed that Rab10, which was listed amongst our 13 predicted targets, was a miR-378a-3p target in the development of esophageal squamous cell carcinoma (Ding et al., 2018). Although the mature sequences of miR-378d and miR-378a-3p have only 86% similarity, their seed sequences are the same. Therefore, Rab10 was selected to investigate further as a most likely target of miR-378d in *M. tb* infection.

**Figure 6:**
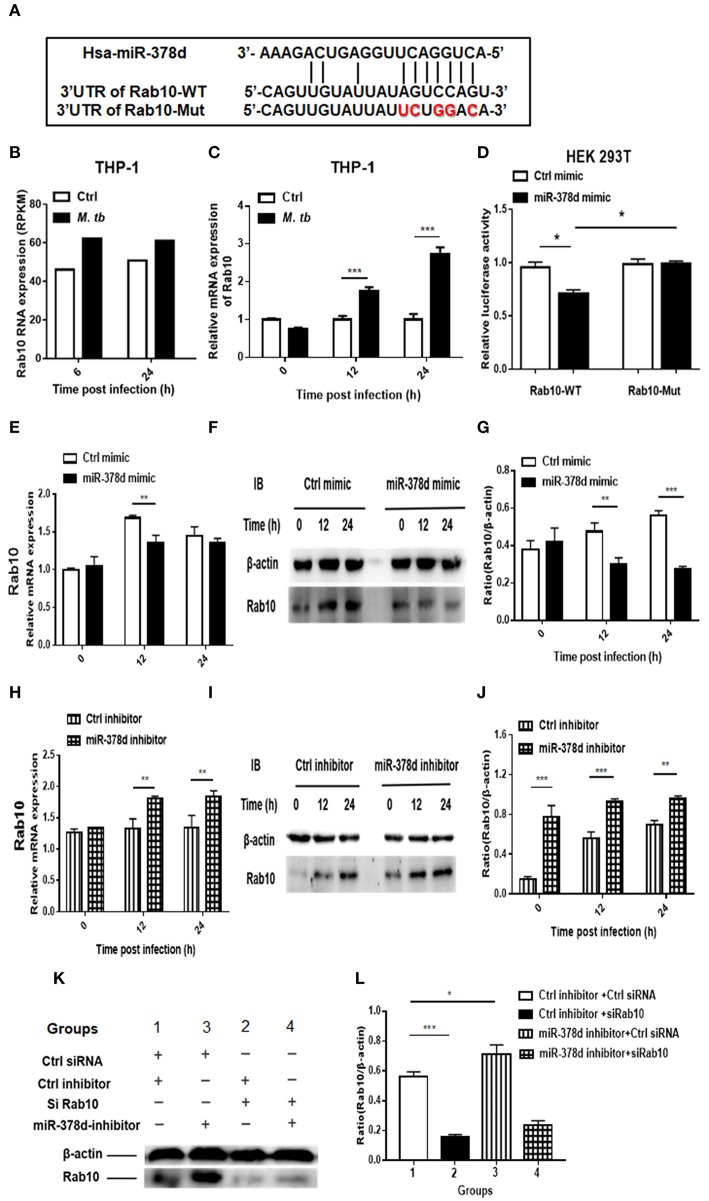
qRT-PCR and Western blot analysis indicating that miR-378d targets Rab10. **(A)** Binding sites between the 3′-UTR of Rab10-WT/Rab10-Mut mRNA and miR-378d as predicted by four online prediction algorithms. **(B)** Expression of Rab10 mRNA in *M. tb*-infected, PMA-differentiated THP-1 cells detected previously by RNA-seq at 6 and 24 h post infection. **(C)** Increased Rab10 expression was confirmed by qRT-PCR in PMA-differentiated THP-1 cells infected with *M. tb* at a MOI of 10 at 12 and 24 h PI compared with the uninfected control. **(D)** The dual luciferase assay demonstrated that miR-378d targeted the 3′-UTR of Rab10-WT but not Rab10-Mut in HEK 293T cells. **(E)** Detection of the regulation of Rab10 mRNA expression by the miR-378d mimic in *M. tb-*infected, PMA-differentiated THP-1 cells using qRT-PCR. **(F)** Detection of the regulation of Rab10 protein expression by miR-378d mimic using western blot assay. **(G)** The intensity of bands produced by western blot assay in **(F)** was quantified using Image J, and the ratio of Rab10/β-actin was calculated and plotted using GraphPad Prism. **(H)** Detection of the regulation of Rab10 mRNA expression by the miR-378d inhibitor in *M. tb-*infected, PMA-differentiated THP-1 cells using qRT-PCR. **(I)** Detection of the regulation of Rab10 protein expression by miR-378d inhibitor using Western blot assay. **(J)** The intensity of bands produced by western blot assay in **(I)** was quantified using Image J, and the ratio of Rab10/β-actin was calculated and plotted using GraphPad Prism. **(K)** Inhibitory effect of siRNA against Rab10 (siRab10) on Rab10 expression and countervailing effects on the miR-378d inhibitor role in increase of Rab10 expression. **(L)** The intensity of bands in the western blot assay **(K)** was quantified using ImageJ, and the ratio of Rab10/β-actin was calculated and plotted using GraphPad Prism. These graphs represent one of three, independent experiments. The *p* < 0.05, 0.01, and 0.001 values were marked by *, **, and ***, respectively.

The bioinformatic analysis showed that there is a high complementary sequence between Rab10 3′-UTR and miR-378d (Figure 6A). By reviewing the previous transcriptional sequence data of this laboratory, we found that Rab10 was up-regulated in *M. tb*-infected THP-1 cells (Figure 6B). This increased expression of Rab10 during *M. tb* infection was validated using qRT-PCR at 12 h and 24 h PI (*p* < 0.001) compared to the uninfected control (Figure 6C). Further, we investigated whether miR-378d interacted with the binding sites at the 3′-UTR of Rab10 mRNA using a luciferase reporter assay in HEK 293T cells. The results showed that the relative luciferase activity in the cells co-transfected with the miR-378d mimic and Rab10-WT was significantly less than the cells co-transfected with control mimic and Rab10-WT in HEK 293T cells (*p* < 0.05). On the other hand, as expected, the luciferase activity did not show differences between cells co-transfected with Rab10-Mut/miR-378d mimic and Rab10-Mut/control mimic (Figure 6D).

Further, the expression of Rab10 was examined by transfecting THP-1 cells with miR-378d mimic or inhibitor and subsequently infecting them with *M. tb*. As shown in Figure 6E, Rab10 mRNA expression was significantly decreased at 12 h PI in cells transfected with miR-378d mimic compared to control mimic (*p* < 0.01), but not at 0 and 24 h PI. When cells were treated with miR-378d inhibitor, the mRNA expression of Rab10 was significantly increased at 12 and 24 h PI compared to the control inhibitor (*p* < 0.01) (Figure 6H). In addition, at the protein level, the expression of Rab10 was examined using Western blot assay, and Rab10 was confirmed to be significantly decreased when cells were treated with the miR-378d mimic compared to the control mimic at 12 h (*p* < 0.01) and 24 h PI (*p* < 0.001) but not at 0 h (Figures 6F,G). On the contrary, the expression of Rab10 was significantly increased at all three time points, when cells transfected with miR-378d inhibitor were compared to control inhibitor (*p* < 0.01) (Figures 6I,J).

Rab10 expression was then examined in THP-1 cells co-transfected with siRab10 and miR-378d inhibitor, control inhibitor, or control siRNA. As shown in Figures 6K,L, Rab10 was expressed at a significantly higher level when cells were treated with miR-378d inhibitor compared to the control inhibitor (Group 3 vs. 1) (*p* < 0.05). When Rab10 was knocked-down with siRNA (siRab10), Rab10 expression was decreased to 27.8% of the control (Group 2 vs. 1) (*p* < 0.001). Notably, when Rab10 was silenced by siRab10, there was no difference in Rab10 expression between with the group co-transfected with miR-378d inhibitor and inhibitor control (Group 4 vs. 2) (*p* > 0.05) (Figures 6K,L). Together, our data demonstrated that miR-378d targets Rab10.

### Inhibition of miR-378d Increased Cytokine Production Through Rab10

**Figure 7:**
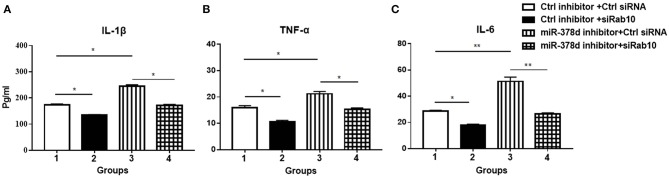
miR-378d inhibitor increased cytokine production through Rab10 in THP-1 cells infected with *M. tb*. THP-1 cells were co-transfected with siRab10 and miR-378d inhibitor, control inhibitor, or control siRNA for 24 h, treated with PMA for 12 h, and then infected with *M. tb* at a MOI of 10. The concentrations of IL-1β **(A)**, TNF-α **(B)**, and IL-6 **(C)** were detected by ELISA at 24 h post infection. These graphs represent one of three independent experiments. The *p* < 0.05 and 0.001 values were marked by * and **, respectively.

To investigate whether miR-378d regulates cytokine production through Rab10, THP-1 cells were co-transfected with siRab10 and miR-378d inhibitor, control inhibitor, or control siRNA for 24 h, treated with PMA for 12 h, and infected with *M. tb* at the indicated MOI. The concentrations of IL-1β, IL-6, and TNF-α in culture supernatants were detected at 24 h PI. The results showed that compared with the negative control (control inhibitor and siRNA), the miR-378d inhibitor significantly increased production of IL-1β, IL-6, and TNF-α (Group 3 vs. 1) (*p* < 0.05), while siRab10 significantly decreased cytokine secretion (Group 2 vs. 1) (*p* < 0.05). Notably, the increasing effect of miR-378d inhibitor on cytokine production was significantly neutralized by siRab10, and thereby, there was no difference in IL-1β, IL-6, and TNF-α concentrations between the groups treated with miR-378d inhibitor plus siRab10 and siRab10 alone (Group 4 vs. 2) (*p* > 0.05) (Figures 7A–C). These data indicated that miR-378d inhibition increased cytokine production of IL-1β, IL-6, and TNF-α through the increase in Rab10 expression in response to *M. tb* infection.

### Inhibition of miR-378d Reduced *M. tb* Intracellular Survival via Rab10

**Figure 8:**
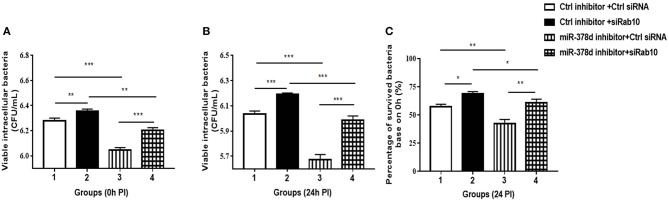
Rab10 decreased *M. tb* survival in macrophages after miR-378d inhibition. **(A,B)** Intracellular *M. tb* numbers in transfected and infected THP-1 cells. THP-1 cells were co-transfected with siRab10 and miR-378d inhibitor, control inhibitor, or control siRNA for 24 h, treated with PMA for 12 h. and infected with *M. tb* at a MOI of 10. At 0 h and 24 h PI, the numbers of intracellular bacteria (CFU/mL) were determined by 7H11 agar plating assay. **(C)** The percentage of survived intracellular *M. tb* at 24 h relative to 0 h. **Group 1**: the control; **Group 2**: Rab10 was knocked-down by siRab10; **Group 3**: miR-378d inhibitor transfection; **Group 4**: co-transfection of miR-378d inhibitor and siRab10. This graph represents one of three independent experiments. The *p* < 0.05, 0.01, and 0.001 values were marked by *, **, and ***, respectively.

To investigate further whether the effect of miR-378d upregulation on the increase in *M. tb* survival is realized through its target Rab10, THP-1 cells were transfected with either siRab10 or miR-378d inhibitor, or co-transfected with both siRab10 and miR-378d inhibitor, with control inhibitor or control siRNA co-transfected when necessary. Then, the transfected THP-1 cells were treated with PMA for 12 h then infected with *M. tb*. The intracellular bacteria were counted by plating assay at 0 h (Figure 8A) and 24 h (Figure 8B) PI. When the Rab10 gene was knocked-down by siRab10, the intracellular *M. tb* number was significantly increased compared to control siRNA at 0 and 24 h PI (Group 2 vs. 1, *p* < 0.01). In contrast, the miR-378d inhibitor significantly decreased the number of intracellular *M. tb* (Group 3 vs. 1, *p* < 0.001) at 0 and 24 h PI. Meanwhile, the co-transfection of miR-378d inhibitor with siRab10 (Group 4) canceled the effect of the miR-378d inhibitor alone on intracellular *M. tb* number (Group 3), leading to a significant increase in CFUs (Group 4 vs. 3, *p* < 0.001) at both 0 and 24 h PI, but decreased when compared to cells treated with inhibitor control and siRab10 (Group 4 vs. 2, *p* < 0.05) (Figures 8B,C).

Consistent with the previous results in Figure 2D, when compared to the initial infection numbers of *M. tb* at 0 h, the percentage of survived intracellular bacteria in Group 3 was the lowest when miR-378d was inhibited, while that in Group 2 was the highest when Rab10 was silenced. Further, the percentage of intracellular bacteria decrease caused by miR-378d inhibitor (Group 3 vs. 1, *p* < 0.01) was reversed by silencing of Rab10 (Group 4 vs. 3, *p* < 0.01), the recovery reach 88.7% of the level of group 2with treatment of miR-378d inhibitor probably although there is still significant difference between them. This is likely because Rab10 expression was only 72.2% of knockdown (Group 4 vs. 2, *p* < 0.01) (Figure 8C). Collectively, *M. tb* intracellular survival could be significantly decreased by inhibition of miR-378d but remarkably increased by both inhibition of miR-378d and knock-down of Rab10. This indicates that it is Rab10 expression that inhibits *M. tb* survival.

## Discussion

So far, quick, effective chemotherapy for TB does not exist, as shown by the long course of treatment (over 6 months) with several drugs combined and a high rate of drug resistance. One of the reason for this is that *M. tb*, a successful intracellular pathogen, can survive and persist for a long term in the hostile microenvironment of host cells, mainly macrophages, by employing mechanisms to escape innate and adaptive immune surveillance (Cosma et al., 2003; Cambier et al., 2014a,b; Dey and Bishai, 2014). On the other hand, host acceptance to bacterial components indicates existence of some essential regulatory mechanisms against *M. tb* infection (Cambier et al., 2014a). One of them is associated with miRNAs, which are crucial post-transcriptional regulators of host immune response (Das et al., 2016). In this study, we have demonstrated that miR-378d plays an important role miR-378d was demonstrated to play a critical role in *M. tb*-infected macrophages through its target, Rab10, and findings contributed to elucidation of the mechanism underlying miRNA activity in host defense or *M. tb* pathogenesis during *M. tb* infection.

### Down-Regulation of miR-378d by *M. tb* Infection Enhanced Inflammation Response to Clear *M. tb*

As introduced previously, miR-378 family members are reported to be induced or inhibited in some tumors and cancers to regulate autophagy (Li et al., 2018b), apoptosis (Costantino et al., 2016; Kuang et al., 2016; Lei et al., 2018; Li et al., 2018b; Ma et al., 2019), cell migration (Ma et al., 2014; Kuang et al., 2016; Templin et al., 2017; Ho et al., 2018), cell proliferation, etc. (Zhang et al., 2017b, 2019; Li et al., 2018a). However, the role for miR-378 in *M. tb* infection has yet to be described.

In this study, miR-378d expression was first demonstrated to be significantly decreased with *M. tb* infection in THP-1 macrophages. Then, we showed that the miR-378d inhibitor decreased, but a miR-378d mimic increased, intracellular *M. tb* numbers. Further experiments discovered that the difference in the intracellular *M. tb* numbers with either miR-378d mimic or inhibitor transfection mainly originated from initial invasion stage, which was defined as 0 h PI in current study, although the co-culture of THP-1 and *M. tb* actually lasted for 12 h for sufficient adhesion and subsequent internalization of the *M. tb*. Similar findings have been reported that *M. tb* infection downregulated miR-20a-5p and induces macrophage apoptosis to enhance *M. tb* clearance through targeting JNK2 in human macrophages (Zhang et al., 2016).

In addition, at late stages of infection (12 and 24 h PI), although the intracellular bacterial numbers between the miR-378d mimic or inhibitor group and the control maintained differences to various degrees, they gradually decreased at similar rates except for the miR-378d mimic treated group at 24 h PI. Therefore, inhibited miR-378d mainly decreased *M. tb* invasion at the early stage of *M. tb* infection in THP-1 macrophages.

### The miR-378d Regulation of *M. tb* Intracellular Survival Was Achieved by Targeting Rab10

In our study, Rab10 was first predicted to be a target of miR-378d using bioinformatics analysis, and then confirmed by a series of experiments at the mRNA and protein levels including complementarity of seed sequences, dual luciferase assay of interaction between Rab10 and miR-378d, measurement of cytokine production, and examination of *M. tb* intracellular survival after knock-down of Rab10 by siRNA (siRab10), with or without miR-378d inhibitor. This finding is in agreement with the previous report that Rab10 is the target of miR-378a-3p (Ding et al., 2018), another miR-378 family member, in esophageal squamous cell carcinomas. Furthermore, the results from the above experiments also demonstrated that the effect of the miR-378d inhibitor on increase of cytokine expression and decrease of intracellular bacterial survival was realized by upregulating its target Rab10. They are in agreement with the previous report that Rab10 promoted production of TNF-α, IL-6, and IFN-β (Wang et al., 2010), and increased the bactericidal activity of bone marrow-derived macrophages during the infection with *Salmonella typhimurium*, another intracellular bacterium (Liu et al., 2017). Besides, Rab10 might decrease *M. tb* survival through promoting phagolysosome fusion (Zerial and McBride, 2001).

### miR-378d Regulation Are Mediated by NF-κB Signaling

Among the four signaling pathways we examined, only interaction between miR-378d and the NF-κB signal pathway was consistently confirmed by different experiments. Down-regulaton of miR-378d significantly activiated NF-κB signaling since expression of P-p65 was increased by miR-378d inhibitor in *M. tb* infected THP-1 cells at all three time points post infection. Meanwhile, this study demonstrated at both the mRNA and protein levels that miR-378d mimic decreased but the inhibitor facilitated production of cytokines IL-1β, TNF-α, and IL-6 in *M. tb* infected THP-1 cells via Rab10. These results are in agreement with a previous publication (Zhang et al., 2017a), which reported that increase of these three cytokines in macrophages induced by *M. tb* infection relied on activation of TLR2/MyD88/NF-κB (Gu et al., 2017). Therefore, there would be a direct but negative regulatory relationship from miR-378d expression to NF-κB signal pathway.

On the other hand, the inhibitor of NF-κB, SC514, significantly neutralized the inhibitory effect of *M. tb* infection on miR-378d expression in THP-1 cells. Therefore, there might be some negative interaction from NF-κB signaling to miR-378d.

Furthermore, the relationship between NF-κB activation and Rab10 expression was previously observed in *Salmonella typhimurium*-infected bone marrow-derived macrophages, in which NF-κB activation was accompanied by enhancement of Rab10 expression and bactericidal activity (Liu et al., 2017).

### miR-378d Regulates Both Macrophages and *M. tb*

Like other miR-378 family members, miR-378d has direct effect on cell proliferation and function. This was supported by our observation in Figure 6D where the difference in expression of Rab10 between the miR-378d mimic and control mimic in cells without *M. tb* infection was significant with *p* < 0.05 (^*^) at 24 h post transfection. However, *M. tb* infection greatly increases the effect of miR-378d on the infected cells. In Figure 6G with *M. tb* expression, the difference in expression of Rab10 between the miR-378d mimic and control mimic in infected cells was very significant with *p* < 0.001(^***^) at 24 h post transfection.

**Figure 9:**
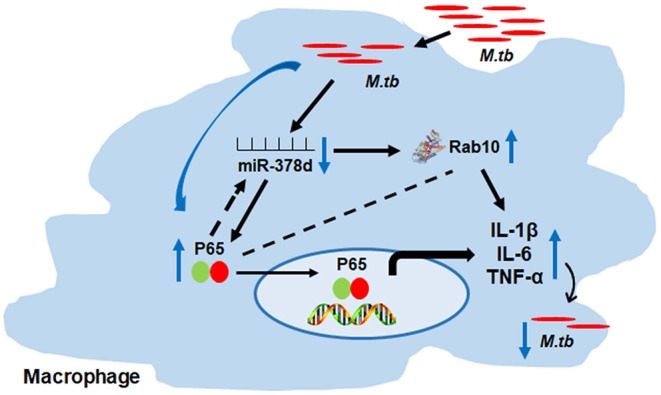
Schema of miR-378d mediated *M. tb* clearance pathway in macrophages. *M. tb* infection may activate the NF-κB signal pathway, then down-regulate the miR-378d expression, subsequently up-regulate the expression of Rab10, prompt the cytokine production and affect *M. tb* survival.

Taken altogether, these findings suggest the possible relationship as follows: *M. tb* infection may activate the NF-κB signal pathway, then down-regulate the miR-378d expression, subsequently up-regulat the expression of its target, Rab10, promptthe above cytokine production and, finally, affect *M. tb* invasion and subsequent intracellular survival (Figure 9).

Regarding to whether medium FBS affected the experiments, although serum in medium is known to influence the macrophage response to different stimuli such as blocking binding of the microorganisms to host cells, we think that FBS shouldn't affect significantly our results and conclusions based on following reasons. Firstly, we were only concerned about the intracellular *M. tb* by adding gentamicin in the medium to kill extracellular *M. tb*; Secondly, we set the un-infected control for the infected experiments to minimize the effect of FBS. Thirdly in natural infection, serum usually co-exists with the bacteria. However, it is worthwhile to study further whether FBS affects efficiency of *M. tb* infection and intracellular survival.

## Conclusion

The Rab10 is the target of miR-378d during *M. tb* infection of THP-1 macrophages. Down-regulation of miR-378d induced by *M. tb* infection significantly enhanced Rab10 expression and decreased *M. tb* survival into the macrophages during *M. tb* infection. The possible mechanism has been proposed involving in activation of NF-κB signaling pathway and thereby induction of pro-inflammatory cytokines. These findings shed a light on further understanding of host defense mechanisms against *M. tb* infection.

## Data Availability Statement

The raw data supporting the conclusions of this article will be made available by the authors, without undue reservation, to any qualified researcher.

## Author Contributions

AG, YZ, and YX contributed conception and design of the study. YZ, YX, HL, DK, and TZ were involved in Bacterial and cell culture studies. YZ, YX, and DK were involved in the construction of the cell infection with *M. tb*. YZ, YX, and DK performed cell transfection, WB and qRT-PCR. YZ, YX, HL, TZ, and DK were involved in bacterial plate assay to count colony-forming units. YZ and XC wrote the first draft of the manuscript. AG revised the manuscript. YC, YP, WZ, CH, HC, JN, and SS participated in the interpretation and discussion of the results. All authors contributed to manuscript, read and approval of the submitted version.

### Conflict of Interest

The authors declare that the research was conducted in the absence of any commercial or financial relationships that could be construed as a potential conflict of interest.

## References

[B1] Alix E. Mukherjee S. Roy C. R. (2011). Subversion of membrane transport pathways by vacuolar pathogens. J. Cell Biol. 195, 943–952. 10.1083/jcb.201105019 22123831 PMC3241728

[B2] Bai X. Feldman N. E. Chmura K. Ovrutsky A. R. Su W. L. Griffin L.. (2013). Inhibition of nuclear factor-kappa B activation decreases survival of Mycobacterium tuberculosis in human macrophages. PLoS ONE 8:e61925. 10.1371/journal.pone.0061925 23634218 PMC3636238

[B3] Bartel D. P. (2004). MicroRNAs: genomics, biogenesis, mechanism, and function. Cell 116, 281–297. 10.1016/S0092-8674(04)00045-5 14744438

[B4] Cambier C. J. Falkow S. Ramakrishnan L. (2014a). Host evasion and exploitation schemes of *Mycobacterium tuberculosis*. Cell 159, 1497–1509. 10.1016/j.cell.2014.11.024 25525872

[B5] Cambier C. J. Takaki K. K. Larson R. P. Hernandez R. E. Tobin D. M. Urdahl K. B.. (2014b). Mycobacteria manipulate macrophage recruitment through coordinated use of membrane lipids. Nature 505, 218–222. 10.1038/nature12799 24336213 PMC3961847

[B6] Chan E. D. Morris K. R. Belisle J. T. Hill P. Remigio L. K. Brennan P. J.. (2001). Induction of inducible nitric oxide synthase-NO* by lipoarabinomannan of Mycobacterium tuberculosis is mediated by MEK1-ERK, MKK7-JNK, and NF-kappaB signaling pathways. Infect. Immun. 69, 2001–2010. 10.1128/IAI.69.4.2001-2010.2001 11254551 PMC98123

[B7] Cheloufi S. Dos Santos C. O. Chong M. M. Hannon G. J. (2010). A dicer-independent miRNA biogenesis pathway that requires Ago catalysis. Nature 465, 584–589. 10.1038/nature09092 20424607 PMC2995450

[B8] Chua C. E. L. Tang B. L. (2018). Rab 10-a traffic controller in multiple cellular pathways and locations. J. Cell. Physiol. 233, 6483–6494. 10.1002/jcp.26503 29377137 PMC13482492

[B9] Cosma C. L. Sherman D. R. Ramakrishnan L. (2003). The secret lives of the pathogenic mycobacteria. Annu. Rev. Microbiol. 57, 641–676. 10.1146/annurev.micro.57.030502.091033 14527294

[B10] Costantino S. Paneni F. Lüscher T. F. Cosentino F. (2016). MicroRNA profiling unveils hyperglycaemic memory in the diabetic heart. Eur. Heart J. 37, 572–576. 10.1093/eurheartj/ehv599 26553540

[B11] Das K. Garnica O. Dhandayuthapani S. (2016). Modulation of host miRNAs by intracellular bacterial pathogens. Front. Cell. Infect. Microbiol. 6:79. 10.3389/fcimb.2016.00079 27536558 PMC4971075

[B12] Dey B. Bishai W. R. (2014). Crosstalk between *Mycobacterium tuberculosis* and the host cell. Semin. Immunol. 26, 486–496. 10.1016/j.smim.2014.09.002 25303934 PMC4250340

[B13] Ding N. Sun X. Wang T. Huang L. Wen J. Zhou Y. (2018). miR-378a-3p exerts tumor suppressive function on the tumorigenesis of esophageal squamous cell carcinoma by targeting Rab10. Int. J. Mol. Med. 42, 381–391. 10.3892/ijmm.2018.3639 29693138 PMC5979826

[B14] Etna M. P. Sinigaglia A. Grassi A. Giacomini E. Romagnoli A. Pardini M.. (2018). *Mycobacterium tuberculosis*-induced miR-155 subverts autophagy by targeting ATG3 in human dendritic cells. PLoS Pathog. 14:e1006790. 10.1371/journal.ppat.1006790 29300789 PMC5771628

[B15] Ganesan J. Ramanujam D. Sassi Y. Ahles A. Jentzsch C. Werfel S.. (2013). MiR-378 controls cardiac hypertrophy by combined repression of mitogen-activated protein kinase pathway factors. Circulation 127, 2097–2106. 10.1161/CIRCULATIONAHA.112.000882 23625957

[B16] Gu X. Gao Y. Mu D. G. Fu E. Q. (2017). MiR-23a-5p modulates mycobacterial survival and autophagy during *Mycobacterium tuberculosis* infection through TLR2/MyD88/NF-κB pathway by targeting TLR2. Exp. Cell Res. 354, 71–77. 10.1016/j.yexcr.2017.03.039 28327409

[B17] Ho C. S. Noor S. M. Nagoor N. H. (2018). MiR-378 and MiR-1827 regulate tumor invasion, migration and angiogenesis in human lung adenocarcinoma by targeting and, respectively. J. Cancer. 9, 331–345. 10.7150/jca.18188 29344280 PMC5771341

[B18] Hutagalung A. H. Novick P. J. (2011). Role of Rab GTPases in membrane traffic and cell physiology. Physiol. Rev. 91, 119–149. 10.1152/physrev.00059.2009 21248164 PMC3710122

[B19] Karim A. F. Chandra P. Chopra A. Siddiqui Z. Bhaskar A. Singh A.. (2011). Express path analysis identifies a tyrosine kinase Src-centric network regulating divergent host responses to *Mycobacterium tuberculosis* infection. J. Biol. Chem. 286, 40307–40319. 10.1074/jbc.M111.266239 21953458 PMC3220550

[B20] Kim J. K. Kim T. S. Basu J. Jo E. K. (2017). MicroRNA in innate immunity and autophagy during mycobacterial infection. Cell. Microbiol. 19:e12687. 10.1111/cmi.12687 27794209

[B21] Kuang X. Wei C. Zhang T. Yang Z. Chi J. Wang L. (2016). miR-378 inhibits cell growth and enhances apoptosis in human myelodysplastic syndromes. Int. J. Oncol. 49, 1921–1930. 10.3892/ijo.2016.3689 27633496

[B22] Kumar M. Sahu S. K. Kumar R. Subuddhi A. Maji R. K. Jana K.. (2015). MicroRNA let-7 modulates the immune response to *Mycobacterium tuberculosis* infection via control of A20, an inhibitor of the NF-kappaB pathway. Cell Host Microbe 17, 345–356. 10.1016/j.chom.2015.01.007 25683052

[B23] Lei X. Zhang B. D. Ren J. G. Luo F. L. (2018). Astragaloside suppresses apoptosis of the podocytes in rats with diabetic nephropathy via miR-378/TRAF5 signaling pathway. Life Sci. 206, 77–83. 10.1016/j.lfs.2018.05.037 29792879

[B24] Li N. Du T. Yan Y. Zhang A. Gao J. Hou G.. (2016). MicroRNA let-7f-5p inhibits porcine reproductive and respiratory syndrome virus by targeting MYH9. Sci. Rep. 6:34332. 10.1038/srep34332 27686528 PMC5043385

[B25] Li W. Liu Y. Yang W. Han X. Li S. Liu H.. (2018a). MicroRNA-378 enhances radiation response in ectopic and orthotopic implantation models of glioblastoma. J. Neurooncol. 136, 63–71. 10.1007/s11060-017-2646-y 29081036 PMC5922763

[B26] Li Y. Jiang J. Liu W. Wang H. Zhao L. Liu S.. (2018b). microRNA-378 promotes autophagy and inhibits apoptosis in skeletal muscle. Proc. Natl. Acad. Sci. U. S. A. 115, E10849–E10858. 10.1073/pnas.1803377115 30373812 PMC6243236

[B27] Liu F. Chen J. Wang P. Li H. Zhou Y. Liu H.. (2018). MicroRNA-27a controls the intracellular survival of *Mycobacterium tuberculosis* by regulating calcium-associated autophagy. Nat. Commun. 9:4295. 10.1038/s41467-018-06836-4 30327467 PMC6191460

[B28] Liu J. Xiang J. Li X. Blankson S. Zhao S. Cai J.. (2017). NF-κB activation is critical for bacterial lipoprotein tolerance-enhanced bactericidal activity in macrophages during microbial infection. Sci. Rep. 7:40418. 10.1038/srep40418 28079153 PMC5227741

[B29] Liu S. da Cunha A. P. Rezende R. M. Cialic R. Wei Z. Bry L.. (2016). The host shapes the gut microbiota via fecal microRNA. Cell Host Microbe 19, 32–43. 10.1016/j.chom.2015.12.005 26764595 PMC4847146

[B30] Ma J. Lin J. Qian J. Qian W. Yin J. Yang B.. (2014). MiR-378 promotes the migration of liver cancer cells by down-regulating Fus expression. Cell. Physiol. Biochem. 34, 2266–2274. 10.1159/000369669 25562172

[B31] Ma J. Wu D. Yi J. Yi Y. Zhu X. Qiu H.. (2019). MiR-378 promoted cell proliferation and inhibited apoptosis by enhanced stem cell properties in chronic myeloid leukemia K562 cells. Biomed. Pharmacother. 112:108623. 10.1016/j.biopha.2019.108623 30797151

[B32] Ma Y. Wu Y. Chen J. Huang K. Ji B. Chen Z.. (2018). miR-10a-5p promotes chondrocyte apoptosis in osteoarthritis by targeting HOXA1. Mol. Ther. Nucleic Acids. 14, 398–409. 10.1016/j.omtn.2018.12.012 30731321 PMC6365368

[B33] Ouimet M. Koster S. Sakowski E. Ramkhelawon B. van Solingen C. Oldebeken S.. (2016). *Mycobacterium tuberculosis* induces the miR-33 locus to reprogram autophagy and host lipid metabolism. Nat. Immunol. 17, 677–686. 10.1038/ni.3434 27089382 PMC4873392

[B34] Pfeffer S. R. (2013). Rab GTPase regulation of membrane identity. Curr. Opin. Cell Biol. 25, 414–419. 10.1016/j.ceb.2013.04.002 23639309 PMC3729790

[B35] Rajaram M. V. S. Ni B. Dodd C. E. Schlesinger L. S. (2014). Macrophage immunoregulatory pathways in tuberculosis. Semin. Immunol. 26, 471–485. 10.1016/j.smim.2014.09.010 25453226 PMC4314327

[B36] Ren Y. Qiu L. Lü F. Ru X. Li S. Xiang Y.. (2016). TALENs-directed knockout of the full-length transcription factor Nrf1α that represses malignant behaviour of human hepatocellular carcinoma (HepG2) cells. Sci. Rep. 6:23775. 10.1038/srep23775 27065079 PMC4827396

[B37] Shen X. Sun W. Shi Y. Xing Z. Su X. (2015). Altered viral replication and cell responses by inserting microRNA recognition element into PB1 in pandemic influenza A virus (H1N1) 2009. Mediators Inflamm. 2015:976575. 10.1155/2015/976575 25788763 PMC4350627

[B38] Stanley S. A. Barczak A. K. Silvis M. R. Luo S. S. Sogi K. Vokes M.. (2014). Identification of host-targeted small molecules that restrict intracellular *Mycobacterium tuberculosis* growth. PLoS Pathog. 10:e1003946. 10.1371/journal.ppat.1003946 24586159 PMC3930586

[B39] Templin C. Volkmann J. Emmert M. Y. Mocharla P. Müller M. Kraenkel N.. (2017). Increased proangiogenic activity of mobilized CD34+ progenitor cells of patients with acute ST-segment-elevation myocardial infarction: role of differential microRNA-378 expression. Arterioscler. Thromb. Vasc. Biol. 37, 341–349. 10.1161/ATVBAHA.116.308695 28062497

[B40] Vallejo A. N. Pogulis R. J. Pease L. R. (2008). PCR mutagenesis by overlap extension and gene SOE. CSH Protoc. 2008:pdb.prot4861. 10.1101/pdb.prot4861 21356760

[B41] Wang D. Lou J. Ouyang C. Chen W. Liu Y. Liu X.. (2010). Ras-related protein Rab10 facilitates TLR4 signaling by promoting replenishment of TLR4 onto the plasma membrane. Proc. Natl. Acad. Sci. U. S. A. 107, 13806–13811. 10.1073/pnas.1009428107 20643919 PMC2922283

[B42] Wang J. X. Zhang X. J. Li Q. Wang K. Wang Y. Jiao J. Q.. (2015). MicroRNA-103/107 regulate programmed necrosis and myocardial ischemia/reperfusion injury through targeting FADD. Circ. Res. 117, 352–363. 10.1161/CIRCRESAHA.117.305781 26038570

[B43] World Health O. (2019). Global Tuberculosis Report 2019. Geneva: World Health Organization.

[B44] Xie X. Lu J. Kulbokas E. J. Golub T. R. Mootha V. Lindblad-Toh K.. (2005). Systematic discovery of regulatory motifs in human promoters and 3' UTRs by comparison of several mammals. Nature 434, 338–345. 10.1038/nature03441 15735639 PMC2923337

[B45] Xu G. Wang J. Gao G. F. Liu C. H. (2014). Insights into battles between Mycobacterium tuberculosis and macrophages. Protein Cell 5, 728–736. 10.1007/s13238-014-0077-5 24938416 PMC4180456

[B46] Yang T. Ge B. (2018). miRNAs in immune responses to *Mycobacterium tuberculosis* infection. Cancer Lett. 431, 22–30. 10.1016/j.canlet.2018.05.028 29803788

[B47] Zeng M. Zhu L. Li L. Kang C. (2017). miR-378 suppresses the proliferation, migration and invasion of colon cancer cells by inhibiting SDAD1. Cell. Mol. Biol. Lett. 22:12. 10.1186/s11658-017-0041-5 28725241 PMC5514464

[B48] Zerial M. McBride H. (2001). Rab proteins as membrane organizers. Nat. Rev. Mol. Cell Biol. 2, 107–117. 10.1038/35052055 11252952

[B49] Zhang G. Liu X. Wang W. Cai Y. Li S. Chen Q.. (2016). Down-regulation of miR-20a-5p triggers cell apoptosis to facilitate mycobacterial clearance through targeting JNK2 in human macrophages. Cell Cycle 15, 2527–2538. 10.1080/15384101.2016.1215386 27494776 PMC5026815

[B50] Zhang T. Hu J. Wang X. Zhao X. Li Z. Niu J.. (2019). MicroRNA-378 promotes hepatic inflammation and fibrosis via modulation of the NF-κB-TNFα pathway. J. Hepatol. 70, 87–96. 10.1016/j.jhep.2018.08.026 30218679 PMC6554744

[B51] Zhang X. Zuo X. Yang B. Li Z. Xue Y. Zhou Y.. (2014). MicroRNA directly enhances mitochondrial translation during muscle differentiation. Cell 158, 607–619. 10.1016/j.cell.2014.05.047 25083871 PMC4119298

[B52] Zhang Z. M. Zhang A. R. Xu M. Lou J. Qiu W. Q. (2017a). TLR-4/miRNA-32-5p/FSTL1 signaling regulates mycobacterial survival and inflammatory responses in *Mycobacterium tuberculosis*-infected macrophages. Exp. Cell Res. 352, 313–321. 10.1016/j.yexcr.2017.02.025 28215633

[B53] Zhang Z. Y. Zhu B. Zhao X. W. Zhan Y. B. Bao J. J. Zhou J. Q.. (2017b). Regulation of UHRF1 by microRNA-378 modulates medulloblastoma cell proliferation and apoptosis. Oncol. Rep. 38, 3078–3084. 10.3892/or.2017.5939 28901497

