# Supplementary material for: Down-Regulation of miR-378d Increased Rab10 Expression to Help Clearance of Mycobacterium tuberculosis in Macrophages
Source: Front Cell Infect Microbiol. 2020 Mar 17;10:108. doi: 10.3389/fcimb.2020.00108 (PMC7094154; doi:10.3389/fcimb.2020.00108)
Supplement: Supplementary file 1 [file Data_Sheet_1.docx]

Supplementary Material

## Supplementary Figures

**
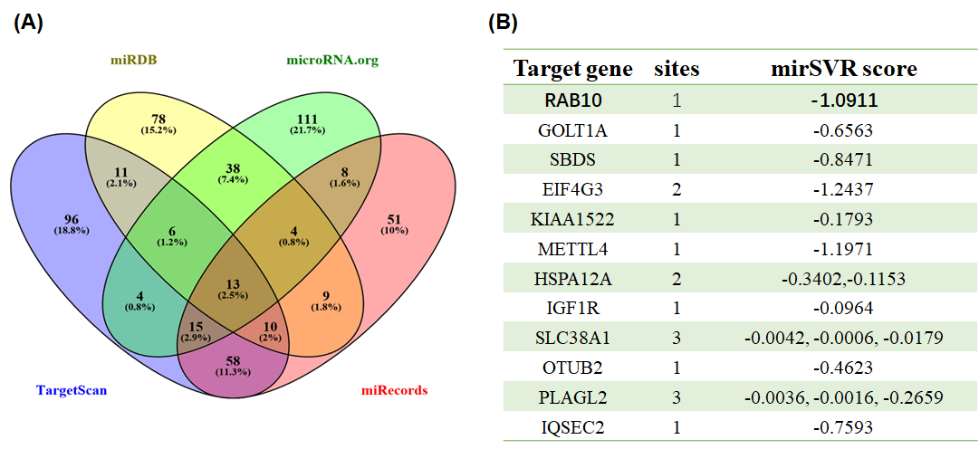
**

**Supplementary Figure 1.** **The target genes of miR-378d predict by four online prediction algorithms. (A)** The target genes of miR-378d predict by four online prediction algorithms shown in venn graph. **(B)** Total of 13 genes could predict by all these four online prediction algorithms.
